# Vocal Attractiveness Matters: Social Preferences in Cooperative Behavior

**DOI:** 10.3389/fpsyg.2022.877530

**Published:** 2022-05-26

**Authors:** Junchen Shang, Zhihui Liu

**Affiliations:** ^1^Department of Medical Humanities, School of Humanities, Southeast University, Nanjing, China; ^2^School of Psychology, Liaoning Normal University, Dalian, China

**Keywords:** vocal attractiveness, cooperative behavior, trust game, ERPs, beauty premium

## Abstract

Research has shown the phenomenon that “what sounds beautiful is good” is a stereotype. It is not clear whether vocal attractiveness affects social decision-making in economic games. Using a modified trust game task, we investigated the neural mechanism of the influence of vocal attractiveness on cooperative decision-making. Participants first heard the voice (attractive or unattractive) of the partner. They had enough time to decide whether to cooperate with the partner for a chance to earn monetary rewards. The behavioral results showed that participants made more invest choices in the attractive partner condition, and they were more likely to cooperate with the female partners in the unattractive voice condition. The event-related potential (ERP) analysis for voice stimuli showed that attractive voices induced larger N1 amplitude than unattractive voices only in the male voice condition. And female voices elicited smaller N1 and larger P2 amplitudes than male voices in both the attractive and unattractive voices condition. A larger P3 amplitude was evoked by female voices and attractive voices. In addition, a more positive late positive complex (LPC) was induced by male voices and attractive voices. This study suggested that attractive voices facilitated cooperative behavior, providing evidence for the “beauty premium” effect of the attractive voices. Moreover, participants were more likely to cooperate with female partners. In the early stage, gender information and male vocal attractiveness were processed automatically, suggesting that male vocal attractiveness was processed preferentially than the female voice. In the late stage, participants allocated attention to both male and female vocal attractiveness.

## Introduction

Attractiveness plays an important role in human evolution and social interactions. Human beings yearn to become beautiful and are willing to choose beautiful people to be their mates. For example, it is well-documented that facial beauty is associated with important benefits, such that people with beautiful faces have obvious advantages in mate selection, job hunting, election campaign, and other social and economic activities (Hamermesh and Biddle, 1994; Watkins and Johnston, 2000; Rhodes, 2006; Ma et al., 2015). This phenomenon is the “what is beautiful is good” stereotype (Dion et al., 1972). In daily life, faces often appear together with voices. The human voice and face are important media for conveying social information. Therefore, beauty is not only in the eyes but also in the ears of the beholder. Feinberg (2008) suggested that both facial and vocal attractiveness are associated with traits indicative of sex hormone levels. Thus, people would combine the information from faces and voices in order to assess mate value (see Groyecka et al., 2017 for a review). From a broader perspective, an attractive voice can also elicit the “what is beautiful is good” stereotype. Individuals with more attractive voices were rated as more likable and dominant (Zuckerman and Driver, 1989). Tigue et al. (2012) found that men with attractive voices were more popular with voters and were more likely to win votes in political elections. Vocal attractiveness has generated growing interest within the academic community. However, the number of research is nowhere near as numerous as the voluminous studies on facial attractiveness (Groyecka et al., 2017).

Acoustic parameters, namely, fundamental frequency (F0) and formant harmonics to noise ratio (HNR), are important clues to evaluate vocal attractiveness (Pisanski and Rendall, 2011). Knowles and Little (2015) found that pitch traits (F0: fundamental frequency; F0-SD: pitch variation) of the female voice and formant traits (Df: formant dispersion; Pf: formant position) of the male voice were positively correlated with cooperativeness ratings. In economic and mating-related contexts, voice pitch was associated with trustworthiness (O’Connor and Barclay, 2017). Trust is one of the most important benefits, which is related to facial attractiveness (Póvoa et al., 2020). Two recent studies (Shtudiner and Klein, 2020; Klein and Shtudiner, 2021) indeed discovered that physical attractiveness and gender influenced moral judgments on workplace behavior. Participants were more tolerant toward attractive people than plain-looking people for unethical work behavior. The impact of attractiveness on women was stronger than on men. To some degree, this study speculated that vocal attractiveness could influence individuals’ trust behavior.

Studies have found that facial attractiveness plays an important role not only in mate selection but also in cooperation behavior. Hamermesh and Biddle (1994) suggested that attractive people had an advantage in the labor market, which they called the “beauty premium” phenomenon. There is ample evidence that supports the “beauty premium,” such that people are more likely to cooperate with the financial partners with attractive faces in economic games (e.g., Mulford et al., 1998; Pandey and Zayas, 2021). For example, attractive partners would be offered more money in an ultimatum game (Solnick and Schweitzer, 1999). In a trust game, trustors gave more money to female trustees who wore makeup to increase their facial attractiveness (Póvoa et al., 2020). In addition, a recent field study shows that attractiveness also has an impact on a consumer environment (Ruffle et al., 2022). When asking for a discount at produce markets, attractive female buyers were offered larger and more frequent discounts than unattractive female buyers, while facial attractiveness did not impact the discount offered to male buyers. Many neurophysiological studies have demonstrated the temporal stages of neural processing of facial attractiveness in economic decision-making (e.g., Ma and Hu, 2015; Ma et al., 2017). For example, in a trust game (Chen et al., 2012), participants saw the faces of partners and were asked to decide whether to cooperate with the partners for a chance to get rewards. If the partners got investments, they could keep all monetary rewards or return half of the money to the participants. The results showed that unattractive faces elicited larger P2 amplitudes, reflecting the automatic and rapid processing of facial attractiveness. The attractive faces elicited larger N2 amplitudes, suggesting that the enhanced attention toward attractive faces is because of their biological significance. However, Jin et al. (2017) found different results in an online peer-to-peer lending task in which the faces of female borrowers were first presented, then male participants (lenders) decided whether to lend ¥1,000 or ¥5,000, followed by feedback on whether the borrowers could repay on time. The results showed that attractive faces induced a smaller N2 component. This may suggest that men are more sensitive to attractive female faces, which may elicit more expectation and attention.

The feedback-related negativity (FRN) component is also one of the important components in the economic decision-making. Ma et al. (2017) found that unfair offers elicited more negative FRN than fair offers in the unattractive-face condition during the ultimatum game, whereas fairness did not influence FRN in the attractive-face condition. In a trust game (Chen et al., 2012), the FRN difference wave (loss minus gain feedback) elicited by the feedback stimuli was larger in the attractive-face condition compared to the unattractive-face condition. The FRN difference wave indicated that participants had a higher expectation that attractive partners would return the monetary rewards compared to unattractive partners. It was not clear whether there was an effect of vocal attractiveness on FRN in trust decision-making.

Some studies have found that facial attractiveness ratings are positively correlated with vocal attractiveness ratings (Saxton et al., 2009; Hughes and Miller, 2016). Facial attractiveness and vocal attractiveness signify similar mating preferences (Groyecka et al., 2017). In the labor market, individuals with attractive voices also had certain advantages. Vocal attractiveness can predict conscientiousness and agreeableness in job performance (Degroot and Kluemper, 2007). Are the effects of vocal attractiveness and facial attractiveness on cooperative behavior similar? In other words, is there a “beauty premium” for voice in cooperative behavior? This question is important since there are many real-world examples of voice-only interaction. A lot of people listen to the broadcast. There are also salespeople who call our cell phones to promote their products. Moreover, people often communicate by voice using software such as WeChat. Some employers also interview employees on the telephone in the labor market. In these circumstances, vocal attractiveness is a clue that may relate to the trustworthiness and affect cooperation behaviors, especially in economic investments. This study explored whether vocal attractiveness plays an important role in a trust game and its neural mechanism.

Recently, some studies were conducted on the neural mechanism of vocal attractiveness processing using event-related potentials (ERPs). In an implicit tone detection task and an explicit voice attractiveness judgment task, Zhang et al. (2020) found that attractive male voices induced a larger N1 because attractive male voices might be a signal indicating mate value and dominance. Moreover, a smaller P2 was elicited by attractive male voices compared with unattractive male voices. Attractive voices elicited larger P3 amplitudes than unattractive voices. Besides, female voices induced larger P2 and P3 amplitudes than male voices. These results indicate that individuals can quickly perform early attention allocation and acoustic coding on vocal attractiveness information. Also, vocal gender information would be automatically extracted in the early stages. In the explicit task, attractive voices evoked larger LPCs (the late positive complex) than unattractive voices. But no significant attractiveness effect was found in the implicit task. Thus, this study used a modified trust game (Chen et al., 2012) to investigate the influence of vocal attractiveness on cooperative behavior. Given the lack of neurological studies on vocal attractiveness, ERPs were recorded for the voice stimuli in order to investigate their neural mechanisms.

In addition, previous studies have also found that decision-making may be affected by the gender of partners. Nonetheless, the results were not consistent. Some studies suggested that people were more likely to cooperate with female partners and allocate more money to them (Solnick, 2001; Fabre et al., 2015). Fabre et al. (2015) thought more positive and emphatic feelings were induced by female proposers. However, another study found that the money investment did not differ according to the gender of partners (Buchan et al., 2008). The gender of partners was identified by their names in the above three studies. Another study found that people allocated more money to male partners when their faces were presented (Solnick and Schweitzer, 1999). However, when the partner’s face was untrustworthy, people were more likely to accept offers from female partners (Wu et al., 2018). Moreover, when the lower-pitched voice of a partner was presented, participants were more likely to cooperate with male partners on money investment (O’Connor and Barclay, 2017). The various findings and controversies in the literature might be caused by different stimuli and different decision-making contexts. It is still not clear whether the effect of vocal attractiveness on people’s cooperative behavior would be modulated by voice gender.

In sum, this study explored whether the processing of vocal attractiveness and voice gender in the trust game occurs in the early stages of processing. A large portion of vocal attractiveness studies are based on behavioral data. To the best of our knowledge, this study is the first to test the neural processing of vocal attractiveness in economic games in voice-only interaction using ERPs. Only one ERPs study provides evidence of neural processing of vocal attractiveness based on a perceptual task and a rating task (Zhang et al., 2020). We provide further evidence for the beauty premium of voices in a more natural setting. We hypothesized that attractive voices may also induce the “beauty premium” effect similar to attractive faces, even if the voices were only presented for 400 ms. Besides, Shtudiner and Klein (2020) figured out that the beauty premium was stronger for female accountants compared to male accountants. Therefore, this study hypothesized that individuals would be more willing to invest in partners with attractive voices, and that voice gender would also affect cooperative behavior. In addition, we analyzed participants’ ERPs during voice presentation to provide neural markers for processing vocal attractiveness. We wished to show that the ERP pattern elicited by vocal attractiveness was similar to Zhang et al. (2020).

## Materials and Methods

### Participants

A total of 64 students (28 females, *M*_age_ = 21.59 years, *SD* = 2.86 years) from Liaoning Normal University were recruited. They all had normal hearing and normal or corrected to normal vision. All participants were physically and mentally healthy. The experiment was approved by the Research Ethics Committee of Liaoning Normal University. Informed consent was obtained from each participant before the experiment. Each participant received a certain reward after the experiment for their participation.

### Design and Materials

The study adopted a 2 (vocal attractiveness: attractive, unattractive) × 2 (voice gender: female, male) within-subjects design, with vocal attractiveness and voice gender as within-participant variables. The dependent variable was the ratio of investment.

The voice stimuli were selected by Zhang et al. (2020), with 160 voice samples (80 female voices and 80 male voices), including five neutral vowel syllables (/a/,/ai/,/ao/,/ei/,/ou/). The duration of all voice samples is equalized to 400 ms, using Praat software (version 5.3.85).^1^ The intensity of all sounds is 70 dB. The neutral vowels were used as experimental stimuli because they can make participants perceive the attractiveness of voices without being affected by irrelevant variables such as emphasis or semantic meaning. Moreover, it is easier to calculate the acoustic parameters of vowels than words and sentences (Ferdenzi et al., 2013). In total, 60 participants (30 males, *M*_age_ = 21.42 years, *SD* = 2.41 years) who did not participate in the ERP experiment were recruited to evaluate the vocal attractiveness of the voices on a 7-point scale ranging from 1 (very unattractive) to 7 (very attractive).

According to the mean ratings of vocal attractiveness, 40 female voices (20 attractive voices and 20 unattractive voices) and 40 male voices (20 attractive voices and 20 unattractive voices) were selected for the ERP experiment. The descriptive statistics of the vocal attractiveness ratings are shown in Table 1. A two-way ANOVA was performed on the attractiveness ratings. The main effect of vocal attractiveness was significant, *F*_(1, 76)_ = 1105.07, *p* < 0.001, η_p_^2^ = 0.94, 95% CI = [0.91, 0.95]. The main effect of voice gender was not significant, *F*_(1, 76)_ = 0.17, *p* = 0.684. The interaction between voice gender and vocal attractiveness was not significant, *F*_(1, 76)_ = 3.24, *p* = 0.076.

**TABLE 1:** Means and standard deviations of vocal attractiveness ratings.

	Male voices		Female voices	
	*M*	*SD*	*M*	*SD*
Attractive voices	5.34	0.40	5.23	0.34
Unattractive voices	2.65	0.28	2.82	0.34

*The ratings of vocal attractiveness were based on a 7-point scale (1 = very unattractive; 7 = very attractive).*

The Praat software was used to analyze the acoustic parameters of attractive and unattractive voices. As shown in Table 2, F0, f1, and Pf were higher in unattractive male voices than in attractive male voices. The jitter of attractive male voices was significantly higher than unattractive male voices. F0, f3, and HRN in unattractive female voices were significantly lower than in attractive female voices. Moreover, the f1 of attractive female voices was significantly lower than unattractive female voices. Previous studies have shown that lower-pitched male voices are more attractive (Collins, 2000; Feinberg et al., 2005; Jones et al., 2010; Re et al., 2012), and higher-pitched female voices are more attractive (Feinberg et al., 2008; Zheng et al., 2020). The differences in acoustic parameters between attractive and unattractive voices in this study were approximately in line with previous studies, suggesting that vocal attractiveness is affected by acoustic parameters. The acoustic parameters of male and female voices were also separately compared in attractive and unattractive conditions, as shown in Table 3. F0, f1, f3, f4, Df, Pf, and HNR in attractive female voices were higher than in attractive male voices. The shimmer in attractive female voices was lower than in attractive male voices. In addition, F0, f2, and Pf in unattractive female voices were higher than in unattractive male voices.

**TABLE 2:** Acoustic differences between attractive and unattractive voices.

Female voices						Male voices				
	Attractive voices	Unattractive voices	*t*	*p*	Cohen’s *d*	Attractive voices	Unattractive voices	*t*	*p*	Cohen’s *d*
F0	256.64 (20.42)	224.03 (22.06)	**4.85**	**<0.001**	1.53	118.28 (10.16)	161.91 (21.08)	**−8.34**	**<0.001**	−2.64
f1	636.51 (170.87)	769.03 (223.48)	−**2.11**	**0.042**	−0.67	494.67 (143.25)	700.14 (161.13)	−**4.26**	**<0.001**	−1.35
f2	1529.96 (602.79)	1867.93 (497.88)	−1.93	0.061	−0.61	1596.63 (531.75)	1412.38 (462.00)	1.17	0.249	0.37
f3	3160.15 (273.57)	2974.21 (245.77)	**2.26**	**0.030**	0.72	2839.67 (158.45)	2886.02 (214.96)	−0.78	0.442	−0.25
f4	4098.84 (308.96)	3992.31 (333.72)	1.05	0.301	0.33	3742.21 (150.77)	3832.93 (206.22)	−1.59	0.121	−0.50
Df	1154.11 (121.11)	1074.43 (141.09)	1.92	0.063	0.61	1082.51 (73.70)	1044.27 (87.96)	1.49	0.144	0.47
Pf	0.30 (0.51)	0.35 (0.49)	−0.29	0.771	−0.09	−0.47 (0.27)	−0.18 (0.34)	−**3.07**	**0.004**	−0.97
Jitter	0.68 (0.22)	0.62 (0.23)	0.86	0.397	0.27	0.77 (0.25)	0.58 (0.30)	**2.19**	**0.034**	0.69
Shimmer	4.72 (1.18)	5.61 (2.68)	−1.35	0.185	−0.43	6.12 (1.65)	5.91 (1.51)	0.41	0.681	0.13
HNR	17.75 (2.74)	15.46 (2.42)	**2.80**	**0.008**	0.89	15.84 (2.50)	15.86 (3.53)	−0.02	0.988	−0.01

*Means are shown, and standard deviations are given in parentheses. The bold values mean p < 0.05.*

*F0, fundamental frequency in Hz; f1 to f4, formant frequencies in Hz; Df, formant dispersion in Hz; Pf, formant position; Jitter, variation of the pitch in μs; Shimmer, variation of energy in dB; HNR, harmonic-to-noise ratio in dB.*

**TABLE 3:** Acoustic differences between female and male voices.

Attractive voices						Unattractive voices				
	Female voices	Male voices	*t*	*p*	Cohen’s *d*	Female voices	Male voices	*t*	*p*	Cohen’s *d*
F0	256.64 (20.42)	118.28 (10.16)	**27.13**	**<0.001**	8.58	224.03 (22.06)	161.91 (21.08)	**9.11**	**<0.001**	2.88
f1	636.51 (170.87)	494.67 (143.25)	**2.85**	**0.007**	0.90	769.03 (223.48)	700.14 (161.13)	1.12	0.270	0.35
f2	1529.96 (602.79)	1596.63 (531.75)	−0.37	0.713	−0.12	1867.93 (497.88)	1412.38 (462.00)	**3.00**	**0.005**	0.95
f3	3160.15 (273.57)	2839.67 (158.45)	**4.53**	**<0.001**	1.43	2974.21 (245.77)	2886.02 (214.96)	1.21	0.235	0.38
f4	4098.84 (308.96)	3742.21 (150.77)	**4.64**	**<0.001**	1.47	3992.31 (333.72)	3832.93 (206.22)	1.82	0.077	0.58
Df	1154.11 (121.11)	1082.51 (73.70)	**2.26**	**0.030**	0.71	1074.43 (141.09)	1044.27 (87.96)	0.81	0.422	0.26
Pf	0.30 (0.51)	−0.47 (0.27)	**5.97**	**<0.001**	1.89	0.35 (0.49)	−0.18 (0.34)	**3.94**	**<0.001**	1.25
Jitter	0.68 (0.22)	0.77 (0.25)	−1.19	0.243	−0.38	0.62 (0.23)	0.58 (0.30)	0.49	0.626	0.16
Shimmer	4.72 (1.18)	6.12 (1.65)	−**3.08**	**0.004**	−0.98	5.61 (2.68)	5.91 (1.51)	−0.45	0.658	−0.14
HNR	17.75 (2.74)	15.84 (2.50)	**2.30**	**0.027**	0.73	15.46 (2.42)	15.86 (3.53)	−0.41	0.682	−0.13

*Means are shown, and standard deviations are given in parentheses. The bold values mean p < 0.05.*

*F0, fundamental frequency in Hz; f1 to f4, formant frequencies in Hz; Df, formant dispersion in Hz; Pf, formant position; Jitter, variation of the pitch in μs; Shimmer, variation of energy in dB; HNR, harmonic-to-noise ratio in dB.*

### Procedure

Participants were comfortably seated in a sound-attenuated and electrically shielded laboratory. The participants’ chins were fixed on a chin rest. The voices were presented binaurally *via* headphones (Sennheiser, HD201). The volume was individually adjusted for each participant to their comfortable level.

**FIGURE 1:**
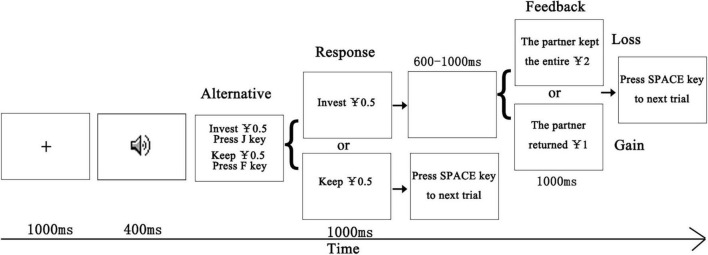
The trial procedure of the trust game task. A central fixation cross was presented for 1,000 ms, followed by a voice of the partner presented for 400 ms. The participants would decide whether to invest ¥0.5 or keep it. The final decision would be presented for 1,000 ms. If the participant agreed to invest, the partner may either give ¥1 back to the participant or keep the entire ¥2. If the participant kept ¥0.5, they would retain the current amount of money.

This experiment employed a modified trust game (Chen et al., 2012). The investment amount and choices were the same as in Chen et al. (2012). Before the experiment, participants were given ¥20. Participants would decide whether to cooperate with “real” partners (represented by attractive or unattractive voices) for a chance to earn monetary rewards (Figure 1). The partners were actually fictional, but participants were not told about this. The amount of money could be accumulated by the investment behavior. The experimental remuneration was related to the final amount of money they owned in the trust game. If the partners returned more money, the participants would get more remuneration. At the beginning of each trial, a central fixation cross was presented for 1,000 ms, followed by the voice of the partner presented for 400 ms. Subsequently, two phases, namely, “invest ¥0.5” and “keep ¥0.5,” were presented on the screen. The participant decided whether to invest ¥0.5 in the partner or keep it. Half of the participants were instructed to press the “F” key if they chose to invest ¥0.5, or to press the “J” key if they refused to invest. The response keys were reversed for the remaining participants. Once their decision had been made, the final decision would be presented for 1,000 ms to emphasize the choice made. If the participant agreed to invest, the partner would receive ¥2. The partner may either give ¥1 back to the participant or keep the entire ¥2. If the participant invested ¥0.5, a blank screen was shown for 600–1,000 ms. Then the participant would see the feedback from the partner, which might be a gain outcome or a loss outcome for 1,000 ms. If the participant kept ¥0.5, they would retain the current amount of money. At the end of each trial, participants were instructed to press the space key to continue. The formal experiment consisted of 480 trials presented in pseudorandom sequence, with half of the trials gaining and the other half of the trials losing. Each voice was present once in each trial. There were 80 voices. In order to retain enough artifact-free ERP trials after artifact rejection, each voice was repeated six times. Half the time, the voices are associated with gains, and the other half the time, they are associated with losses. After the experiment, participants reported that they were not aware of these manipulations and did not know the regularity of investment feedback. There were eight practice trials at the beginning of the experiment for the participants to familiarize the task. The voices in the practice trials were not present in the formal experiment. The feedback in practice trials was not included in the payment.

After the game, the subjects rated the attractiveness of the 80 voices presented in the formal experiment on a 7-point scale (1 = very unattractive; 7 = very attractive). The purpose was to ensure that the perceived attractiveness of the voices was consistent with the different trends of the pre-selected voice ratings.

### Behavioral Data Analysis

The percentage of the number of investment choices for both attractive and unattractive voice conditions was calculated to obtain the ratio of investment. The ratio of investment was analyzed by a 2 (vocal attractiveness: attractive, unattractive) × 2 (voice gender: female, male) repeated measures analysis of variance, with vocal attractiveness and voice gender as within-participant variables.

Since the payoff of participants was accumulated across trials, their decisions of keeping or investing might also be influenced by (i) collected money until the current trial, together with the (ii) decision of the partner from the last trial. In order to explore how these factors (feedback from the last trial, the amount of money until the current trial, vocal attractiveness, and voice gender of the current partner) predict participants’ investment intentions, multiple linear regression models (MLRM) were used to test the factors affecting the ratio of investment.

### Event-Related Potential Recording and Analysis

Electroencephalography (EEG) was recorded by a 64 scalp sites electrode cap (Brain Products, GmbH, Germany). A cephalic (forehead) location was connected as the ground electrode. Two electrodes were separately placed below and on the right side of the participant’s right eye to record the vertical and horizontal electrooculogram (EOG). The sampling rate was 500 Hz. All electrode impedances were maintained below 5 kΩ. The recorded EEGs were offline, re-referenced to the average of the left and right mastoids.

The EEG data were analyzed using the Brain Vision Analyzer Version 2 (Brain Products, GmbH, Germany). EOG artifacts were corrected first, followed by digital filtering through a 0.01 Hz high-pass cutoff and a low-pass at 30 Hz (24 dB/octave). Independent component analysis algorithms were used to correct the EOG. ERP trials with EOG artifacts, amplifier clipping artifacts, or peak-to-peak deflections exceeding ± 80 μV were excluded from the final averaging. The EEG data was time-locked to the onset of voice stimuli. The EEGs were segmented for 1,200 ms into epochs initiated at 200 ms before and 1,000 ms after the stimulus onset. The EEG epochs were averaged separately for the attractive female/male voices and unattractive female/male voices. The data of seven subjects were excluded due to excessive artifacts. There were 58 valid subjects (28 females) remaining. After artifact rejection, there were 99.84 trials in the attractive-female condition, 99.24 trials in the unattractive-female condition, 99.62 trials in the attractive-male condition, and 99.83 trials in the unattractive-male condition.

According to the scalp distribution and the previous studies (Chen et al., 2012; Zhang et al., 2020), we selected the following 15 electrode sites for statistical analysis: F3, FC3, C3, CP3, P3 (5 left sites); Fz, FCz, Cz, CPz, Pz (5 midline sites); and F4, FC4, C4, CP4, P4 (5 right sites). The following time windows were selected to examine the hemisphere and region effects associated with vocal attractiveness processing: N1 (120–170 ms), P2 (170–230 ms), P3 (350–440 ms), and LPC (470–700 ms) components. Mean amplitudes for each component were submitted to a four-way repeated measures analysis of variance (ANOVA) including within-subject variables, namely, vocal attractiveness (attractive and unattractive), voice gender (female and male), region (front, fronto-central, central, central-parietal, and parietal), and hemisphere (left, middle, and right). Results that did not conform to the spherical hypothesis were corrected with the Greenhouse-Geisser method for all analyses.

The average amplitude of the FRN wave (230–310 ms) was measured to investigate the ERP waves elicited by feedback stimuli. According to previous studies (Ma et al., 2015, 2017), FRN amplitudes were submitted to a two-way repeated measures analysis of variance (ANOVA) including the following within-subject factors, namely, vocal attractiveness (attractive and unattractive), feedback (gain and loss), and electrode sites (F3, Fz, F4, FC3, FCz, FC4, C3, Cz, and C4). During the feedback presentation, the EEG epochs were separately averaged for four conditions, namely, attractive voice-gain, unattractive voice-gain, attractive voice-loss, and unattractive voice-loss. In order to ensure that at least 30 valid trials remained in each condition, for the analysis of FRN waves, there were 43 valid subjects (23 female). Results that did not conform to the spherical hypothesis were corrected with the Greenhouse-Geisser method for all analyses.

## Results

### Investment Ratios

**FIGURE 2:**
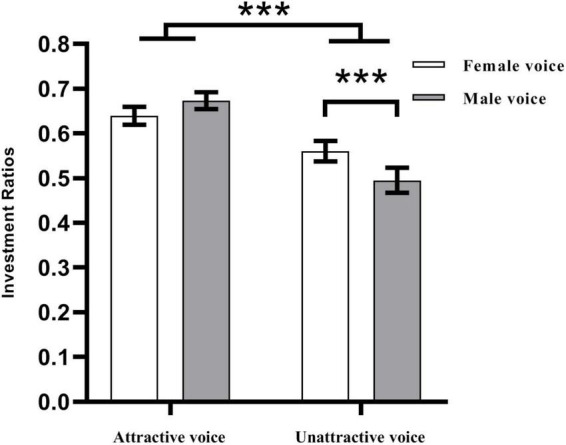
The investment ratio of the participants in four conditions (attractive-female, attractive-male, unattractive-female, and unattractive-male). ***p < 0.001. Error bars represent one standard error about the mean.

The participants were more likely to cooperate with attractive partners (*M* = 0.66, *SD* = 0.16) than unattractive partners (*M* = 0.53, *SD* = 0.20), *F*_(1, 63)_ = 35.81, *p* < 0.001, η_p_^2^ = 0.36, 95% CI = [0.18, 0.51]. There was no significant main effect for voice gender, *F*_(1, 63)_ = 1.36, *p* = 0.247. The interaction effect between vocal attractiveness and voice gender was significant, *F*_(1,63)_ = 16.10, *p* < 0.001, η_p_^2^ = 0.20, 95% CI = [0.05, 0.36]. In unattractive voice condition, participants were more likely to invest to female partners (*M* = 0.56, *SD* = 0.18) compared to male partners (*M* = 0.50, *SD* = 0.22), *p* < 0.001. Whether the partners were male or female, attractive partners would get more investment, *p*-values ≤ 0.001 (Figure 2).

In the regression model, the amount of money until the current trial was not significantly related to investment ratios (β = 0.042, *t* = 1.23, *p* = 0.221). Vocal attractiveness and voice gender both made a significant contribution to the prediction of investment ratios (β = 0.652, *t* = 18.93, *p* < 0.001; β = 0.072, *t* = 2.08, *p* = 0.038, respectively). Feedback from the last trial also made a significant contribution to the prediction of investment ratios (β = −0.090, *t* = −2.59, *p* = 0.010). Vocal attractiveness, voice gender, and feedback of the last trial accounted for 43.6% of investment ratios. The general model framework is as follows:


Y=0.051X+10.015X-2-0.018X+30.337.

Y = predicted value of investment ratios; X_1_ = vocal attractiveness, X_2_ = voice gender, X_3_ = feedback of the last trial.

### Rating Results

Independent sample *t*-test was conducted on attractiveness ratings, the ratings of attractive voices (*M* = 5.10, *SD* = 0.35) were significantly higher than unattractive voices (*M* = 3.08, *SD* = 0.45), *t*(78) = 22.34, *p* < 0.001, Cohen’s *d* = 5.00.

### Event-Related Potential Analysis

**FIGURE 3:**
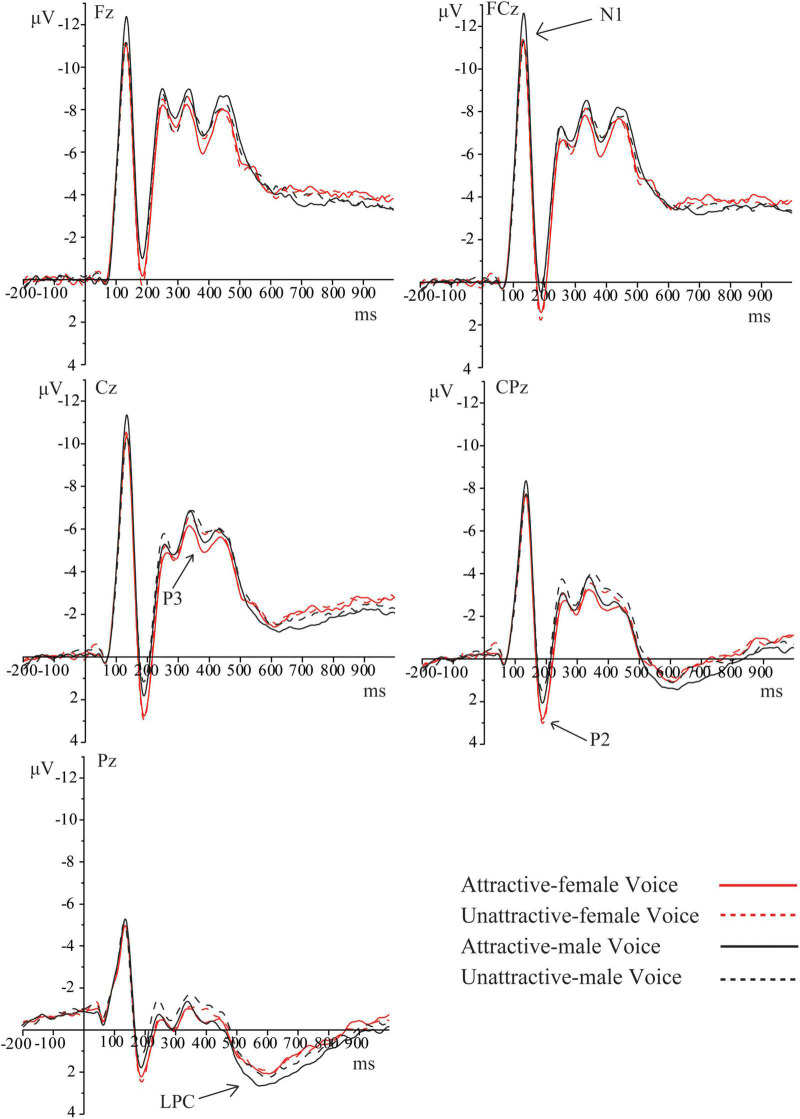
Grand-average ERPs at Fz, FCz, Cz, CPz, and Pz for the four conditions. The time window for N1 was 120–170 ms. The time window for P2 was 170–230 ms. The time window for P3 was 350–440 ms. The time window for LPC was 470–700 ms. ERPs were time-locked to the onset of each voice.

**FIGURE 4:**
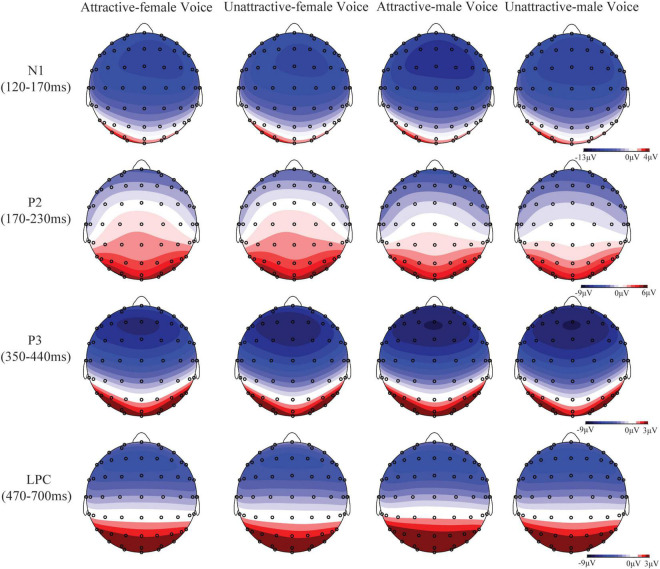
Topography maps of voltage amplitudes for N1, P2, P3, and LPC in the four conditions.

**FIGURE 5:**
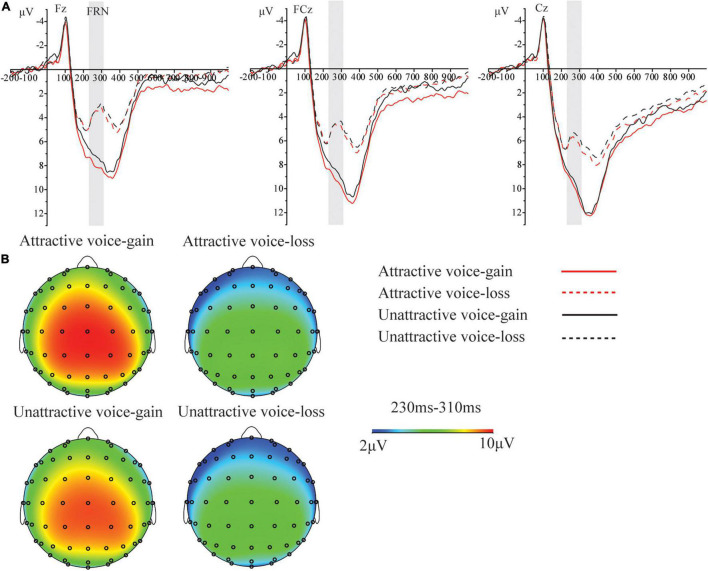
**(A)** Grand average ERPs induced by the feedback of gain and loss at three representative electrodes in the attractive and unattractive voice conditions. **(B)** Topography of scalp distribution and waves generated by gain and loss in the attractive and unattractive voice conditions.

As shown in Figures 3, 4, obvious N1, P2, P3, and LPC components were elicited by attractive male and female voices and unattractive male and female voices. The FRN component elicited by attractive and unattractive voices, and gain and loss are shown in Figure 5.

#### N1 Component (120–170 ms)

There was a significant main effect not only for voice gender, (*F*_(1, 57)_ = 27.93, *p* < 0.001, η_p_^2^ = 0.33, 95% CI = [0.14, 0.48]), but also for vocal attractiveness, (*F*_(1, 57)_ = 5.94, *p* = 0.018, η_p_^2^ = 0.09, 95% CI = [0.002, 0.25]). Besides, there was a significant interaction of vocal attractiveness by gender, hemisphere, and region, *F*_(5.48, 312.11)_ = 2.31, *p* = 0.039, η_p_^2^ = 0.04, 95% CI = [0.00, 0.07]. Analysis of simple effects showed that there was no significant effect on attractiveness in female voice condition. The following significant effects were applied to the male voice. In any hemisphere, attractive voices elicited larger N1 amplitudes in the front, fronto-central, and central regions, with *p*-values < 0.039. Attractive voices elicited larger N1 amplitudes in the central-parietal region of the left hemisphere, *p* = 0.048. In attractive voice condition, male voices elicited larger N1 amplitudes than female voices in the front, fronto-central, central, and central-parietal regions of the left and right hemispheres, with *p*-values < 0.011. Male voices elicited larger N1 amplitudes in any region of the middle hemisphere, with *p*-values < 0.020. In unattractive voice condition, male voices elicited larger N1 amplitudes in the front and fronto-central regions of the left hemisphere, with *p*-values ≤ 0.029. A more negative N1 was elicited by male voices in the fronto-central, central and parietal regions of the middle hemisphere, with *p*-values ≤ 0.036. Male voices elicited larger N1 amplitudes in the front, fronto-central, and central regions of the right hemisphere, with *p*-values ≤ 0.021.

#### P2 Component (170–230 ms)

The main effect of voice gender was significant, *F*_(1, 57)_ = 61.79, *p* < 0.001, η_p_^2^ = 0.52, 95% CI = [0.33, 0.64]. Additionally, there was a significant interaction of attractiveness by voice gender, hemisphere, and region, *F*_(5.66, 322.57)_ = 3.15, *p* = 0.006, η_p_^2^ = 0.05, 95% CI = [0.005, 0.09]. Follow-up analyses revealed that the effect of vocal attractiveness was only applied to male voices. In the middle hemisphere, attractive male voices elicited larger P2 amplitudes in the central-parietal and parietal regions, with *p*-values ≤ 0.049. In the attractive or unattractive voice condition, female voices elicited larger P2 amplitudes in any region of all hemispheres, with *p*-values < 0.047.

#### P3 Component (350–440 ms)

There was a significant main effect not only for voice gender, (*F*_(1, 57)_ = 8.37, *p* = 0.005, η_p_^2^ = 0.13, 95% CI = [0.01, 0.29]), but also for vocal attractiveness, (*F*_(1, 57)_ = 5.80, *p* = 0.019, η_p_^2^ = 0.09, 95% CI = [0.002, 0.25]). The interaction of vocal attractiveness by voice gender and region was significant, *F*_(1.38, 78.59)_ = 5.62, *p* = 0.012, η_p_^2^ = 0.09, 95% CI = [0.005, 0.22]. Analysis of simple effect showed that attractive female voices elicited larger P3 amplitudes than attractive male voices in central, frontal, and fronto-central regions, with *p*-values ≤ 0.013. In unattractive voice condition, female voices elicited a more positive P3 compared with male voices in the parietal region, with *p* = 0.048. In female voice condition, attractive voices elicited a more positive P3 compared with unattractive voices in the central and central-parietal regions, with *p*-values ≤ 0.025. In male voice condition, a more positive P3 was elicited by attractive voices than unattractive voices in the central-parietal and parietal regions, with *p*-values < 0.007.

#### Late Positive Complex Component (470–700 ms)

The interaction of voice gender, hemisphere, and region was significant, *F*_(6.18, 352.33)_ = 2.92, *p* = 0.008, η_p_^2^ = 0.05, 95% CI = [0.004, 0.08]. In the left or right hemisphere, male voices elicited a more positive LPC in the parietal region than female voices, with *p*-values < 0.041. Besides, there was a significant interaction of vocal attractiveness, hemisphere, and region, *F*_(5.29, 301.39)_ = 2.35, *p* = 0.038, η_p_^2^ = 0.04, 95% CI = [0.00, 0.07]. In the left or middle hemisphere, attractive voices elicited a more positive LPC in the parietal region, with *p*-values < 0.014.

#### Feedback-Related Negativity (230–310 ms)

There was no significant effect related to vocal attractiveness. The effect of feedback was significant, *F*_(1, 42)_ = 65.00, *p* < 0.001, η_p_^2^ = 0.61, 95% CI = [0.40, 0.72]. The effect of site was significant, *F*_(2.55, 106.94)_ = 17.08, *p* < 0.001, η_p_^2^ = 0.29, 95% CI = [0.14, 0.40]. The interaction between feedback and the site was significant, *F*_(3.45, 145.02)_ = 12.20, *p* < 0.001, η_p_^2^ = 0.23, 95% CI = [0.10, 0.32]. Analysis of simple effects showed that the feedback of gain elicited a smaller FRN than the feedback of loss in any site, with *p*-values < 0.001.

## Discussion

This study explored the influence of vocal attractiveness on cooperative behavior in a trust game. The behavioral results showed that participants were more likely to invest money in partners with attractive voices. The results provided evidence for the “what sounds beautiful is good” stereotype (Zuckerman and Driver, 1989). People with attractive voices were considered to have more favorable traits (Zuckerman and Driver, 1989; Phil et al., 2014; Rezlescu et al., 2015). People with positive personalities were more likely to be trusted by other people. Besides, these results were similar to that of Chen et al. (2012) who stated that partners with attractive faces received more investments. This study suggested that vocal attractiveness induced the “beauty premium” effect similar to that of facial attractiveness (e.g., Hamermesh and Biddle, 1994; Ma and Hu, 2015; Ma et al., 2017). Facial attractiveness is likely to lead to social and economic benefits such as trust (Póvoa et al., 2020). It is possible that vocal attractiveness is also associated with trust. Therefore, people may think that partners with unattractive voices are more untrustworthy. Moreover, this study also found that participants were more willing to invest money in female partners in the unattractive voice condition. Wu et al. (2018) found that people were more willing to accept a female proposer’s monetary offers in untrustworthy face condition. The findings of this study are similar to that of Wu et al. (2018). Prior research suggested that people showed more prosocial behavior toward women in the ultimatum game. Participants showed a higher willingness to cooperate with female proposers and the acceptance rate of the monetary offers from female proposers was significantly higher than that of the male proposers (Eckel and Grossman, 2001; Fabre et al., 2015). In addition, Ruffle et al. (2022) investigated whether buyer sex and buyer attractiveness could affect shopping discounts by designing a field experiment. Female buyers were able to get more discounts. An attractive appearance also made female buyers enjoy larger discounts. But this phenomenon did not apply to male buyers. Therefore, attractive women tend to have some advantage in economic decision-making activities.

Event-related potential results showed that the significant effect of vocal attractiveness in N1 was only applied to the male voices. Attractive male voices induced larger N1 amplitudes than unattractive male voices. N1 component was associated with the early attention to emotional valence (Ma et al., 2017). It is possible that attractive voices induced positive emotions and induced larger N1 amplitudes. Furthermore, Zhang et al. (2020) also found that vocal attractiveness significantly affected N1 in the implicit (voice un-related) tone detection task and the explicit vocal attractiveness judgment task only in the condition of male voices. This was consistent with the results of this study. A similar result was also found in the early stage of P2, where larger P2 amplitudes were evoked by attractive male voices. In addition, male voices induced larger N1 amplitudes than female voices in both attractive and unattractive conditions, suggesting that participants could quickly process the gender information of voice in the early stage. N1 component mainly reflects the early attention to auditory information (Woldorff et al., 1993; Latinus and Taylor, 2012). In the initial processing stage of voices, participants might mainly judge the voice gender and attractiveness of male voices, resulting in differences in N1 amplitudes.

In addition, female voices elicited larger P2 amplitudes than male voices in the attractive voice condition in the front, fronto-central, and central regions. Female voices induced larger P2 amplitudes in the unattractive voice condition in the parietal region. The results showed that the processing of voice gender dimorphism continued from N1 to P2 stage. Zhang et al. (2020) also pointed out that the cognitive processing of voice was no longer limited to the acoustic parameters of voices. P2 was not only one of the key components to explore the effect of voice recognition (Schweinberger, 2001; Charest et al., 2009) but also a key component of voice gender recognition (Zäske et al., 2009). Latinus and Taylor (2012) also found that female voices induced a larger P2 amplitude in the voice gender discrimination task, which reflected sensitivity to voice gender. Latinus and Taylor dissociated pitch processing and still found larger P2 amplitudes induced by female voices. Therefore, the P2 component mainly reflected the cognitive processing of voice gender information.

Furthermore, female voices evoked larger P3 amplitudes than male voices over the front, fronto-central, and central regions in attractive voice condition. Female voices also evoked larger P3 amplitudes over the parietal region in unattractive voice condition. The P3 effect further revealed the processing of discriminating voice gender (Zäske et al., 2009). In sum, female voices elicited a positive shift of N1, P2, and P3 components compared to male voices. It is possible that the prosocial attitude toward female partners accompanied more cognitive resources for female voices compared to male voices. This is partially consistent with the behavioral results that showed female voices occupied a certain advantage in unattractive voice condition. In female voice condition, attractive voices evoked larger P3 amplitudes in the central and central-parietal regions. In male voice condition, attractive voices evoked larger P3 amplitudes in the central-parietal and parietal regions. These results were consistent with Zhang et al. (2020). The findings illustrated that participants also discriminated vocal attractiveness during the P3 stage. Thus, after 300 ms of voice presentation, attractive voices captured more attention from participants. Besides, larger P3 amplitudes were evoked by attractive faces (Zhang and Deng, 2012; Ma et al., 2017), suggesting that the process of vocal attractiveness is similar to facial attractiveness. The reason may be that attractive voices had a reward effect (Bestelmeyer et al., 2012; Ma et al., 2017), and the attractive voice was associated with positive personalities (Zuckerman et al., 1990; Oguchi and Kikuchi, 1997; Rezlescu et al., 2015). Furthermore, P3 was also associated with the sensitivity of rewards (Yeung and Sanfey, 2004). In this trust game, participants were more willing to invest money in partners with attractive voices. Participants may also expect to get gains from partners with attractive voices, showing larger P3 amplitudes.

This study found that attractive voices elicited more positive LPC, which was consistent with Zhang et al. (2020). In some decision-making tasks, attractive faces induced larger LPPs (late positive potentials) (Ma et al., 2015, 2017). Emotion could have a significant effect on LPP (see Hajcak and Foti, 2020 for a review). Moreover, LPP is highly correlated with motivation (Bradley, 2009). In the trust game, attractive voices may induce positive emotional experience, which cause larger LPCs. According to the behavioral results, participants showed higher investment motivation for partners with attractive voices. We also found that male voices induced a larger LPC. Zhang et al. (2020) also pointed out that the significant effect of gender appeared in the late stage in the rating task of vocal attractiveness. Combined with behavioral results, voice gender could significantly affect participants’ investment decisions. This study indicated that elaborated cognitive processing of voice gender and vocal attractiveness occurred in the late stage in order to prepare for whether to invest or not.

In contrast to previous studies (Chen et al., 2012; Ma et al., 2017), this study did not find that vocal attractiveness had a significant effect on FRN. The possible reason may be that the influence of attractiveness on the decision may not have lasted until the feedback stage because the voice was only presented for 400 ms. Nonetheless, the influence of gain or loss feedback on FRN was much larger than vocal attractiveness. Future research can extend the duration of voice presentation in order to explore if vocal attractiveness affects participants’ cognitive processing of investment feedback.

There were several limitations in this study. First, we adopted the same decision-making task as Chen et al. (2012). Each decision is dichotomous (invest ¥0.5 or nothing) and individually risks only ¥0.5. This amount may not be high enough in 2022 and impact not only the perception of voice but also the evaluation processing of the feedback. Future studies should use more investment choices and higher risk amounts to explore the impact of vocal attractiveness on trust behavior. Second, each subject made 480 decisions, and each voice was presented six times. Since there were 80 voices, and some ERP trials were excluded from analysis after artifact rejection, artifact-free ERP trials would not be enough if each subject listened only to one voice. Future research should use more voice databases and retest the effect of vocal attractiveness when each subject only listens to one voice.

## Conclusion

This study provides further evidence for the neural activity of the processing of vocal attractiveness and the “beauty premium” effect of the attractive voice in cooperative behavior. Partners with attractive voices enjoyed an advantage in the trust game. Individuals also exhibited more prosocial behaviors toward female partners when their voices were unattractive. In the early N1 processing stage, participants had certain advantages in processing male vocal attractiveness. The processing of male vocal attractiveness was an automatic process. Female voices induced smaller N1 and larger P2 and P3 amplitudes than male voices, indicating that voice gender was processed even though it was task-irrelevant at the early stage and captured attention resources at the following P3 stage. At the P3 stage, processing of both female and male vocal attractiveness occurred, reflecting that the processing of vocal attractiveness needed attention allocation. Male voices and attractive voices elicited more positive LPC at the late stage, reflecting that participants refined the information about voice gender and vocal attractiveness.

## Data Availability Statement

The raw data supporting the conclusions of this article will be made available by the authors, without undue reservation.

## Ethics Statement

The studies involving human participants were reviewed and approved by the Research Ethics Committee of Liaoning Normal University. The participants provided their written informed consent to participate in this study.

## Author Contributions

JS and ZL designed the experiments, analyzed the data and wrote the manuscript, and revised the manuscript. ZL prepared the materials and performed the experiments. Both authors contributed to the article and approved the submitted version.

## Conflict of Interest

The authors declare that the research was conducted in the absence of any commercial or financial relationships that could be construed as a potential conflict of interest.

## Publisher’s Note

All claims expressed in this article are solely those of the authors and do not necessarily represent those of their affiliated organizations, or those of the publisher, the editors and the reviewers. Any product that may be evaluated in this article, or claim that may be made by its manufacturer, is not guaranteed or endorsed by the publisher.

## Footnotes

1
http://www.fon.hum.uva.nl/praat/
